# Interrater and intrarater reliability of four different classification methods for evaluating acromial morphology on standardized radiographs

**DOI:** 10.1016/j.jseint.2023.02.004

**Published:** 2023-02-28

**Authors:** Thomas W. Mayntzhusen, Adam Witten, Jens Gramkow, Sanja B. Hansen, Shefali A. Chatterjee, Per Hölmich, Kristoffer W. Barfod

**Affiliations:** a Sports Orthopedic Research Center - Copenhagen (SORC-C), Department of Orthopedic Surgery, Copenhagen University Hospital, Amager-Hvidovre, Denmark; b Department of Radiology, Center for Functional and Diagnostic Imaging and Research, Copenhagen University Hospital Hvidovre, Hvidovre, Denmark

**Keywords:** Acromion, Morphology, Supraspinatus outlet view, Interrater, Intrarater, Reliability, Classification

## Abstract

**Background:**

Acromial morphology is an important pathophysiological factor for the development of subacromial impingement syndrome. There are 3 radiological methods to evaluate acromial morphology: Bigliani, modified Epstein, and acromial angle. However, their reliability have not been compared in a single study, nor using standardized radiographs. Consequently, the evaluation of acromial morphology is currently not validated though its widespread use across the world. The objective of this study was to investigate reliability of the 3 known classifications and the novel Copenhagen Acromial Curve classification.

**Methods:**

Three experienced clinicians rated 102 standardized supraspinatus outlet view radiographs with the 4 classification methods in 2 separate sessions a month apart. All measurements were blinded. With an expected kappa (κ) and intraclass correlation coefficient (ICC) > 0.7 (+/−0.15), the target sample size was 87 radiographs.

**Results:**

The Bigliani classification had interrater and intrarater reliability ranging from fair to good (κ 0.32-0.41 and 0.26-0.62). The modified Epstein classification had fair to good interrater and intrarater reliability (κ 0.24-0.69 and 0.57-0.63). The acromial angle classification had moderate to good interrater and intrarater reliability (κ 0.53-0.60 and 0.59-0.72). The novel Copenhagen Acromial Curve classification showed moderate to good interrater and intrarater reliability (ICC 0.66-0.71 and 0.75-0.78, respectively).

**Conclusion:**

The Copenhagen Acromial Curve was the only classification method with an ICC value > 0.7. The popular Bigliani classification had the worst reliability. The Copenhagen Acromial Curve classification produces numerical data, as opposed to the other 3 classification methods. This could potentially be utilized in future research to establishing cut-off values for treatment stratification.

Subacromial impingement syndrome (SIS) is one of the most frequent shoulder-related disorders.^13^^,^^16^ Acromial morphology is assumed to be an important pathophysiological factor for the development of SIS, and hence alteration of acromial morphology (acromioplasty) is an integrated part of the surgical management of SIS.^8^^,^^28^ Therefore, it is imperative to have a reliable and valid method to classify acromial morphology.

Currently, 3 methods are used to classify acromial morphology^2^^,^^4^^,^^23^^,^^25^^,^^27^; the Bigliani classification, the modified Epstein classification, and the acromial angle classification. In 1986 Bigliani et al described a classification method that qualitatively distinguishes the acromial morphology. The Bigliani classification is the most commonly used, but it has been reported with varying interobserver reliability with kappa (κ) between 0.25 and 0.52.^3^^,^^6^^,^^7^^,^^17^^,^^18^^,^^23^^,^^29^ Subsequent attempts to find a reliable classification method of the acromial morphology has been made by Toivonen et al, who introduced the acromial angle classification in 1995 as a quantitative method of studying the acromial morphology.^25^ The acromial angle classification divides the morphology of the acromion into 3 categories and has shown moderate to good interrater and intrarater reliability.^23^^,^^25^^,^^26^ Another classification method was introduced by Epstein^5^ in 1993, which was later modified by Stehle et al into the ‘modified Epstein classification’.^23^

In past studies, all 3 classification methods have substantial variation in the reported reliability.^3^^,^^6^^,^^7^^,^^17^^,^^18^^,^^23^^,^^29^ More importantly, no study has compared all 3 rating methods in a clinical setting, or using standardized radiographs defined by an objective set of inclusion criteria.

The objective of the study was to investigate the interrater and intrarater reliability of the Bigliani classification, the modified Epstein classification, the acromial angle classification, and the novel Copenhagen Acromial Curve classification. We hypothesized that all classification methods would have a moderate to strong interrater and intrarater reliability, defined as a κ value above 0.7 for the categorical variables and an intraclass correlation coefficient (ICC) value above 0.7 for the continuous variables.

## Materials and methods

The study adheres to the Guidelines for Reporting Reliability and Agreement Studies.^10^ The study was part of a larger project that was registered and approved by the Regional Committee on Health Research Ethics, Copenhagen (H-19025712).

### Sampling method

Supraspinatus outlet views (SOVs) were identified from a consecutive cohort of shoulder patients referred to the place of investigation from September 2020 to August 2021. All included SOVs were in accordance with the Copenhagen Supraspinatus Outlet View Criteria (CSOV criteria) (Supplementary Appendix S1). The CSOV criteria were developed by the project group prior to the present study to allow for standardization of the included SOVs. The aim of the CSOV criteria is to identify true SOVs with optimal visualization of acromial morphology. Patients with fractures in the shoulder girdle or previous surgery were not considered eligible. X-rays with acromial spurs were included. A single X-ray, with a complete calcification of the coracoacromial ligament, was excluded a priori, as this was believed to be a rare anatomical variant, and not an acromial spur.

### Raters

The radiographs were classified by 1 senior orthopedic shoulder surgeon and 2 senior musculoskeletal radiologists. Rater A was a senior orthopedic shoulder surgeon with more than 18 years of clinical experience. Rater B was a senior musculoskeletal radiologist with more than 9 years of clinical experience. Rater C was a senior musculoskeletal radiologist with more than 13 years of clinical experience. The 3 raters were all familiar analyzing SOV in the daily treatment of patients. All raters were blinded to their own and each other’s results.

### Training phase

To ensure a standardized quality of measurements, all the raters were individually instructed in the 4 different acromial classification methods by the author T.W.M., who showed and explained the classification methods until familiarization was achieved using a standardized printed instruction. The raters then performed each measurement 10 times without supervision. Finally, the raters performed each measurement under supervision. If the measurements were not correctly performed the process was repeated.

### Rating procedure

Each radiograph was rated with the 4 classification methods by the 3 raters in 2 separate sessions a month apart. All radiographs were classified in the following order: Bigliani classification, acromial angle classification, Copenhagen Acromial Curve classification, and modified Epstein classification.

### Acromial classification methods

#### Bigliani

**Figure 1:**
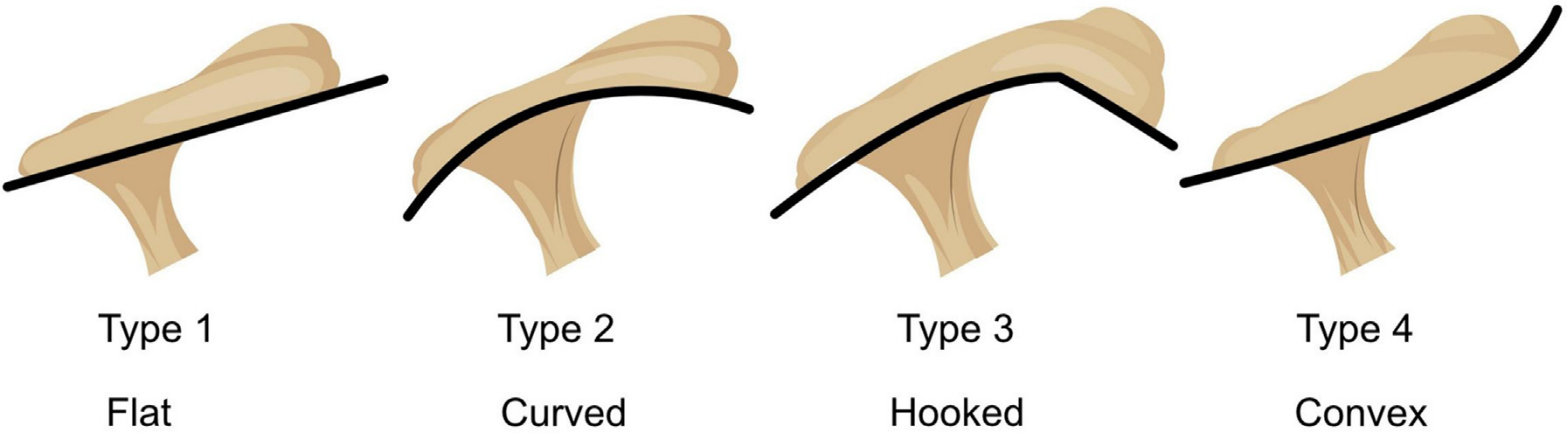
The Bigliani classification described by Bigliani^2^ (figure modified from Bigliani 1991^1^).

The raters visually categorized the acromial morphology as being either flat (type 1), curved (type 2), hooked (type 3), or convex (type 4)^2^^,^^27^ (Fig. 1).

#### Modified Epstein

**Figure 2:**
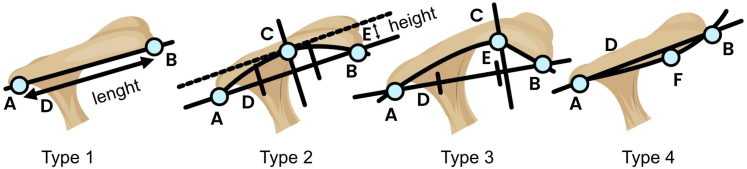
The modified Epstein classification; Type 1: height is less than or equal to 2% of the acromial length. Type 2: highest point (C) is above middle third of the acromial length. Type 3: highest point (C) is above anterior third of the acromial length. Type 4: lowest point of the undersurface (F) is under the acromial length. Point (*A*) and point (*B*) are the most posterior and anterior point of the acromial undersurface, respectively. Point (*C*) is the highest point of the acromial undersurface compared to the acromial length (*D*), which is a straight line connecting point (*A*) and (*B*). Line (*E*) is the acromial height which is defined as a perpendicular from point (*C*) to the acromial length (*D*). Point (*F*) is the lowest point of the undersurface if it is under the acromial length (*D*). The Acromial length (*D*) is divided in to 3 equally large sections.

A line was drawn from the most anterior and most posterior point of the acromial undersurface (acromial length). This line was divided into 3 equally large sections. A second line was drawn perpendicularly to the first line from the highest point on the acromial undersurface (acromial height). The acromial morphology was classified according to the following^23^ (Fig. 2):

• Type 1: Height is less than or equal to 2% of the acromial length.
• Type 2: Height is greater than 2% of the acromial length and the highest point is above the middle third of the acromial length.
• Type 3: Height is greater than 2% of the acromial length and the highest point is above the anterior third of the acromial length.
• Type 4: Lowest point of the undersurface is under the acromial length.

#### Acromial angle

**Figure 3:**
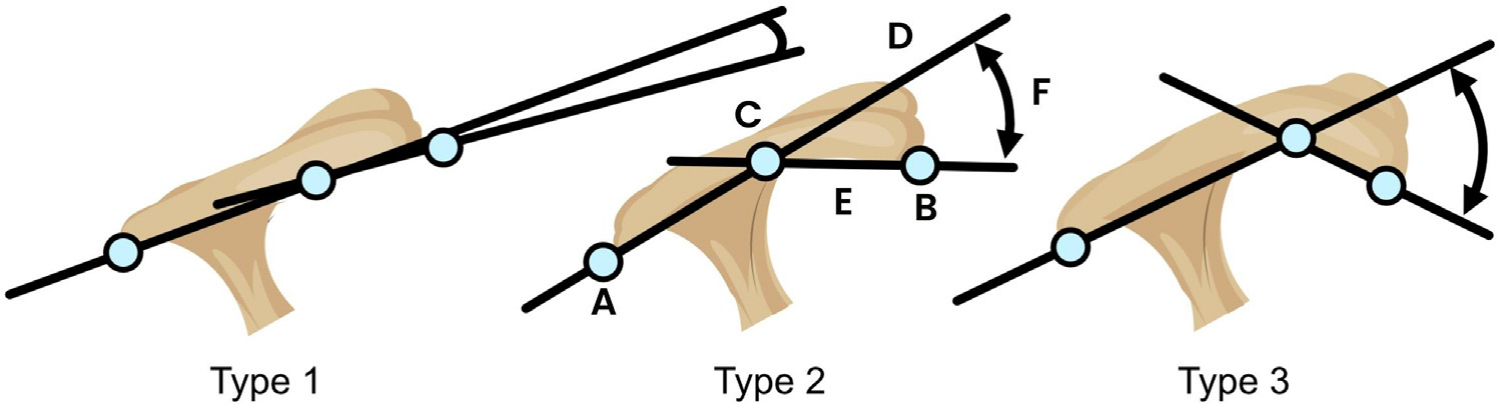
The acromial angle and Copenhagen Acromial Curve classification. The acromial angle classification; Type 1: acromial angle 0-12°, Type 2: 13°–27°, and Type 3: ≥ 28°. Point (*A*) and point (*B*) is the most posterior and anterior point of the acromial undersurface, respectively. Point (*C*) is the highest point of the acromial undersurface compared to a straight line connecting point (*A*) and (*B*). Line (*D*) is a straight line going through point (*A*) and (*C*). Line (*E*) is a straight line going through point (*B*) and (*C*). The angle between line (*D*) and (*E*) is the acromial angle and Copenhagen Acromial Curve.

A line was drawn for the most posterior point of the acromial undersurface to the highest point on the acromial undersurface compared to a straight baseline connecting the most posterior point with the most anterior point of the acromial undersurface. A second line was drawn from the most anterior point of the acromial undersurface to the highest point on the acromial undersurface. Based on the size of the angle the radiographs were classified as being type 1 if the acromial angle was 0°-12°, as type 2 if the angle was 13°-27°, and as type 3 if it was 28° or greater^25^ (Fig. 3).

### Copenhagen Acromial Curve

A line was drawn for the most posterior point of the acromial undersurface to the highest point on the acromial undersurface compared to a straight baseline connecting the most posterior point with the most anterior point of the acromial undersurface. A second line was drawn from the most anterior point of the acromial undersurface to the highest point on the acromial undersurface. The angle between these lines is measured and reported in absolute degrees (Fig. 3).

### Blinding procedure

The radiographs were presented in the computer software IMPAX Client (version 6.7.0.6011 2020; AGFA, Mortsel, Belgium) without patient or image identification. The radiographs were presented in a new randomized order for each classification method. The raters were blinded to their own and the other raters’ results. The first author (T.W.M.) was present during all rating sessions to facilitate the ratings, handing out standardized printed instructions and ensure correct execution of the blinding procedure but did not in any way participate in the ratings.

### Statistics

Interrater and intrarater reliability were assessed using linear weighted κ statistics for categorical variables and ICC 3.1 for continuous variables, respectively. Comparisons between rater A and B, B and C, and C and A were performed separately.

According to the classification of Landis et al, a κ value ≤ 0 was defined as poor, >0-≤0.20 as slight, >0.20-≤0.40 as fair, >0.40-≤0.60 as moderate, >0.60-≤80 as good, and >80 was defined as excellent reliability.^11^ According to the guidelines by Koo and Li ICC values less than 0.5 are indicative of poor reliability, values between 0.5 and 0.75 indicate moderate reliability, values between 0.75 and 0.90 indicate good reliability, and values greater than 0.90 indicate excellent reliability.^9^ Statistics were performed using IBM SPSS Statistics (version 25; IBM Corp., Armonk, NY, USA).

### Sample size calculation

With an expected κ of 0.7 and an expected 95% confidence interval of +/− 0.15, 87 radiographs were needed for the Bigliani, the modified Epstein, and the acromial angle classifications. With an expected reliability (ICC) of 0.7 and an expected 95% confidence interval of +/− 0.15, 46 radiographs were needed for the Copenhagen Acromial Curve classification. The statistical analysis plan and the sample size calculation were developed in cooperation with a senior statistician.

## Results

One Hundred Two SOV from 97 patients were included in this study. Baseline characteristics are shown in Table I. An overview of the 3 raters’ evaluation is presented in Table II.

**Table I:** Baseline characteristics of patients. Categorical data are presented as numbers (percentage). Continuous data are presented as mean (standard deviation).

Variable	Patients
Male sex	41 (40)
Age (yr)	54.7 (14)
Right shoulder	60 (59)

**Table II:** Total assignments (percentage) to the acromial types at first rating for all raters.

	Rater A, B, and C			
Classification method	Type 1	Type 2	Type 3	Type 4
Bigliani	52 (17.0)	188 (61.4)	66 (21.6)	0 (0.0)
Acromial angle	3 (1.0)	139 (45.4)	164 (53.6)	-
Modified Epstein	0 (0.0)	244 (79.7)	62 (20.3)	0 (0.0)

## Discussion

Only the novel Copenhagen Acromial Curve classification showed a moderate to good interrater (ICC 0.66-0.71) and intrarater reliability (ICC 0.75-0.78). This quantitative classification method depends on measuring the same angle as the acromial angle classification; however, the result is reported as numerical data and not ordinal data. Measurement of the angle in the Copenhagen Acromial Curve classification is a fast and easy applicable way of classifying the acromial morphology.

Interestingly the Bigliani classification, which is the most commonly used classification in clinical practice and research, had the lowest interrater reliability with fair to moderate κ values (0.32-0.41) and intrarater reliability ranging from fair to good (0.26-0.62) (Table III). Previous studies have found varying reliability for the Bigliani classification with κ values for intrarater reliability between 0.44-0.89 and interrater reliability between 0.25-0.52 using cadaveric scapulae, magnetic resonance imaging, and radiographs.^3^^,^^6^^,^^7^^,^^17^^,^^18^^,^^23^^,^^29^ The intrarater reliability of the Bigliani classification is surprisingly discouraging with a κ as low as 0.26. As a result, the Bigliani classification cannot be recommended.^18^^,^^23^

**Table III:** Intrarater and interrater kappa and intraclass correlation coefficient values (with 95% confidence intervals) for the 4 acromial classification methods.

	Intrarater reliability			Interrater reliability		
Classification method	Rater A	Rater B	Rater C	Rater A/B	Rater A/C	Rater B/C
Bigliani (Kappa)	0.62 (0.48-0.76)	0.44 (0.28-0.60)	0.26 (0.10-0.42)	0.32 (0.18-0.46)	0.41 (0.28-0.54)	0.40 (0.24-0.57)
Acromial angle (Kappa)	0.59 (0.43-0.74)	0.72 (0.58-0.85)	0.61 (0.46-0.75)	0.60 (0.45-0.76)	0.59 (0.44-0.73)	0.53 (0.37-0.68)
Modified Epstein (Kappa)	0.63 (0.42-0.84)	0.57 (0.38-0.77)	0.61 (0.43-0.79)	0.69 (0.50-0.88)	0.24 (0.03-0.44)	0.26 (0.05-0.47)
Copenhagen Acromial Curve (ICC)	0.75 (0.65-0.82)	0.78 (0.69-0.85)	0.77 (0.65-0.84)	0.66 (0.53-0.76)	0.70 (0.56-0.79)	0.71 (0.60-0.80)

*ICC*, intraclass correlation coefficient.

The acromial angle classification showed a better reliability than the Bigliani classification with κ values 0.53-0.60 for interrater reliability and 0.59-0.72 for intrarater reliability, respectively. However, we found very few type 1 acromions (Table II), which questions whether the cut off points for this classification method are optimal. Previous studies have found quantitative measures like the acromial angle to show fair to good interrater and intrarater reliability.^21^^,^^23^^,^^25^^,^^26^

The modified Epstein classification had a good interrater reliability between raters A/B with a κ of 0.69, but for rater A/C and B/C the reliability was fair with Kappa values of 0.24-0.26. Interestingly rater C had good intrarater reliability with a Kappa value of 0.61, which shows that the modified Epstein classification was interpreted differently between raters. Our finding is in contrast to Stehle et al, who found the modified Epstein to be superior to both the Bigliani and acromial angle classification.^23^ However, the study was performed on a relatively small sample of 24 cadaveric scapulas and might not be generalizable to a clinical population.^23^

The raters in our study had the highest attainable level of education and had many years of experience in evaluating SOVs in clinical treatment of patients. Additionally, the raters went through a standardized training phase of the four classification methods used in the study to ensure optimal measurements.

Analysis of 2-dimensional radiographs of the acromion depends on standardized and optimal SOV since suboptimal radiographs affect the analysis. Especially the acromial hook, which is the angle measured in the Copenhagen Acromial Curve classification, is affected the most by suboptimal radiographs and as little as 5°-10° up- and downward malrotation of the scapula in cranio-caudal direction will change interpretation of the acromial morphology significantly.^22^^,^^24^ To account for this bias only standardized SOVs adhering to the CSOV criteria (Supplementary Appendix S1) were included in the present study. This standardization further distinguishes the present study from previously published clinical studies where no formal evaluation of the SOVs have been performed.^12^^,^^14^^,^^20^ The CSOV criteria are designed to include both radiographs with perfect alignment and a small degree of mal-rotation to reflect radiographs from the clinical every day.

Evaluation of acromial morphology is a difficult task on 2 dimensional x-rays. The acromion is a three dimensional structure with varying curvature in both the frontal and sagittal plane. Many factors affect the results, when classifying a complex three dimensional structure on 2-dimensional x-rays such as raters, standardization of radiographs, and choice of classification method. The results of the present study underline the importance of humility when interpreting acromial morphology based on SOVs. Our results showed that the Copenhagen Acromial Curve classification might be a usable tool to investigate if acromial morphology affects the pathology of the shoulder and future studies should investigate whether it is possible to define clinically relevant cut off points for the Copenhagen Acromial Curve classification.

### Limitations and strengths

A limitation of the study is the interpretation of the results given by the novel Copenhagen Acromial Curve classification, which must be investigated and clarified in future studies. The first author was present during all ratings. Though the instructions were standardized, it may represent an undetected bias. The strengths of this study are the standardization of the SOVs with the CSOV criteria which additionally improves generalizability as it allows the reader to reproduce the setting and compare results between studies using standardized SOVs. This is the first study to investigate the Bigliani, acromial angle, and modified Epstein classification methods in a clinical setting and using standardized radiographs. Furthermore, it introduces the first numeric classification method and the study had a strict randomized setup and blinding procedure.^15^^,^^19^^,^^25^

## Conclusion

The Copenhagen Acromial Curve classification was the only classification method meeting our hypothesis with an ICC value >0.7. The acromial angle classification showed moderate interrater reliability, the modified Epstein classification showed fair to good interrater reliability, and the popular Bigliani classification had the worst reliability. The Copenhagen Acromial Curve classification yields numerical data, as opposed to the other 3 classification methods, and this could potentially be utilized in future research to establishing cut-off values for treatment stratification.

## Disclaimers

Funding: No funding was disclosed by the authors.

Conflicts of interest: The authors, their immediate families, and any research foundation with which they are affiliated have not received any financial payments or other benefits from any commercial entity related to the subject of this article.
