# Supplementary material for: Interrater and intrarater reliability of four different classification methods for evaluating acromial morphology on standardized radiographs
Source: JSES Int. 2023 Feb 28;7(3):445–9. doi: 10.1016/j.jseint.2023.02.004 (PMC10229407; doi:10.1016/j.jseint.2023.02.004)
Supplement: Supplementary Appendix S1 [file mmc1.docx]

## Copenhagen Supraspinatus Outlet View Criteria (CSOV criteria)

The CSOV criteria were developed by the project group prior to the present study to allow for standardization of the included supraspinatus outlet views (SOV’s). The aim of the CSOV criteria was to identify SOV’s with optimal visualization of acromial morphology. To achieve that, it was necessary to project a clear display of the acromial undersurface on X-ray without any double margins. That requires correct rotation of the scapula in both the sagittal, coronal and transverse planes, which we have experienced is obtainable, when a SOV fulfills the CSOV criteria.


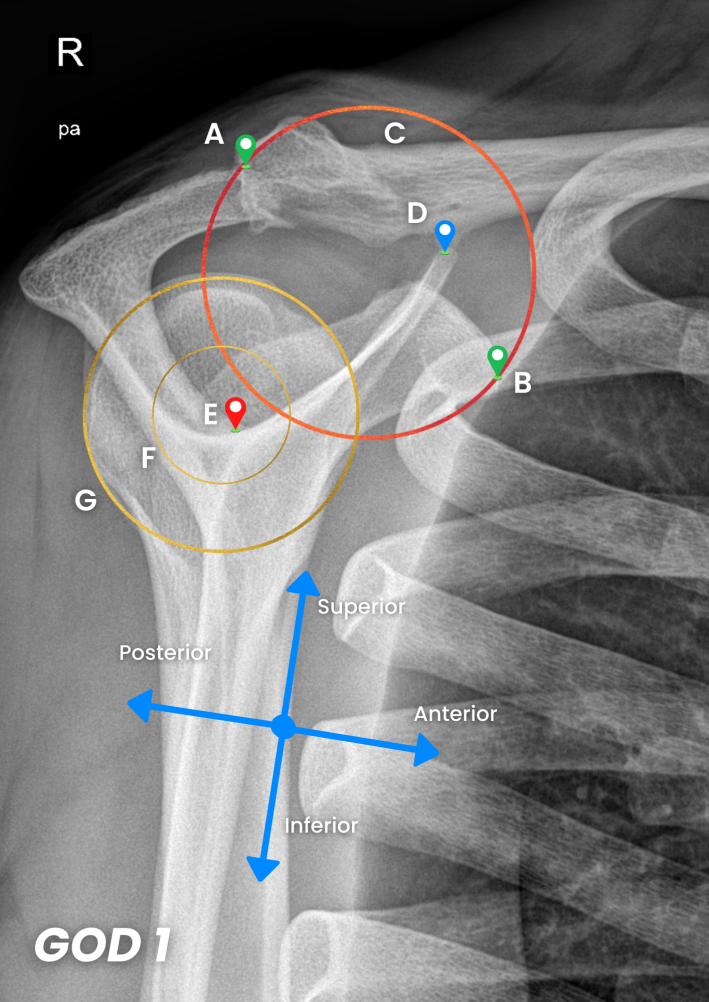


The following anatomical landmarks were used. All landmarks were identified in reference to the longitudinal axis of scapula, defined as the line between angulus superior and angulus inferior:

- Angulus superior (D). The landmark was defined as the most superior point of margo superior.
- Angulus inferior. The landmark was defined as the most inferior point of the scapula
- The anterior coracoid process (B). The landmark was defined as the most anterior point of the coracoid process
- The anterosuperior point of acromion (A). The landmark was defined as the most superior point of the anterior vertical edge of acromion.
- The most inferior point of the fossa supraspinatus (E). If fossa supraspinatus fossa had double margins, the most superior margin was referred to.

The following geometrical shapes were used:

- The acromio-coracoid (AC) circle (C). The circle was drawn with the diameter defined by the anterior point of the coracoid process and the anterosuperior point of acromion.
- The humerus circle (G). The circle was drawn as a best fit circle of the humeral head.
- The small humerus circle (F). The circle was drawn centered in the humerus circle with half the diameter.

CSOV criteria: 1) The angulus superior scapula (D) is within the AC circle (C), and 2) The most inferior point of the supraspinatus fossa (E) is within the half humerus circle (F).

**Development of the CSOV criteria**

*The CSOV criteria allow both SOV with a perfect alignment and a small degree of malrotation to reflect SOV from the clinical every day.*

*In the development process of the CSOV Criteria we evaluated SOV’s from several hundred patients to detect a pattern that differentiates optimal from suboptimal SOV’s as defined subjectively by three experienced shoulder surgeons. They discussed the characterization of an optimal versus suboptimal SOV in plenum based on the large analysis of SOV’s and came to an agreement.*

*Criteria 1 will not be fulfilled if the scapula is malrotated in the craniocaudal or anterior-posterior plane, because the angulus superior will project outside of the designated circle. Furthermore, to double check that there isn’t any malrotation in the craniocaudal plane, which affects the acromial morphology the most, criteria 2 focuses on the supraspinatus fossa which ideally is totally in line. A similar principle was used on cadaveric scapulas by Stehle et al, 2007, where they placed a rod in the fossa supraspinatus and if the scapula was optimally aligned the rod was displayed as a circle on the SOV and the fossa was perfectly aligned. If malrotated in any plane the rod will not display as a circle, but as a 3D cylinder, and the fossa supraspinatus will have double margins on a SOV.*

*The CSOV criteria focuses on the margins of the fossa supraspinatus because on a malrotated SOV, the supraspinatus fossa will appear with double margins and the top margin will be affected the most. Therefore, the most inferior point of the fossa in a malrotated scapula, which refers to the most superior margin if the fossa is not perfectly aligned, will not be in the smaller center circle of the humeral head as described by criteria 2.*


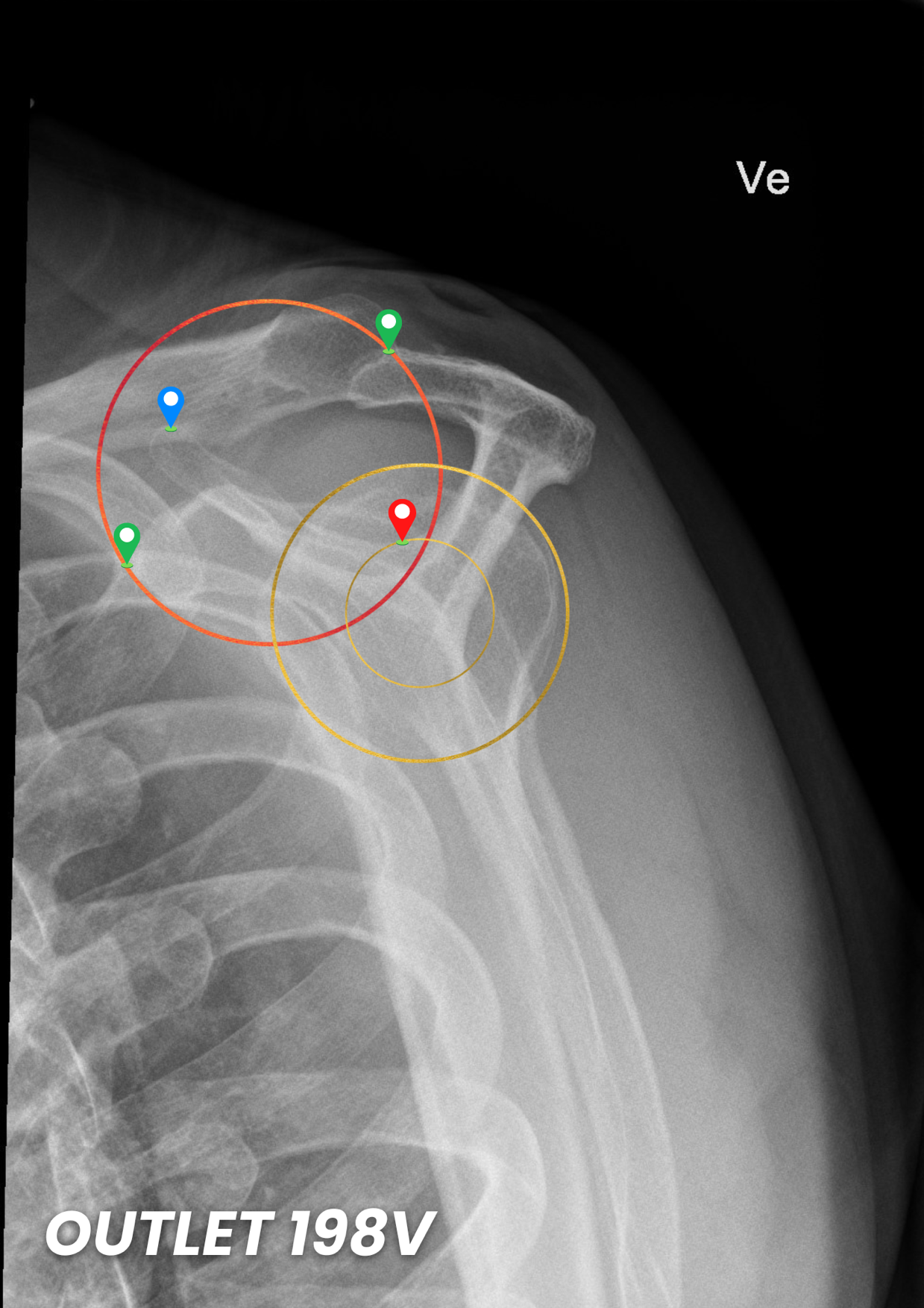
*The following four examples of SOV’s are extremes, which fit within the CSOV criteria.*

**SOV 1**

This scapula has a small amount of craniocaudal malrotation (external rotation), which superimposes the supraspinatus fossa and the most inferior point of the supraspinatus fossa is just inside the half humerus circle.


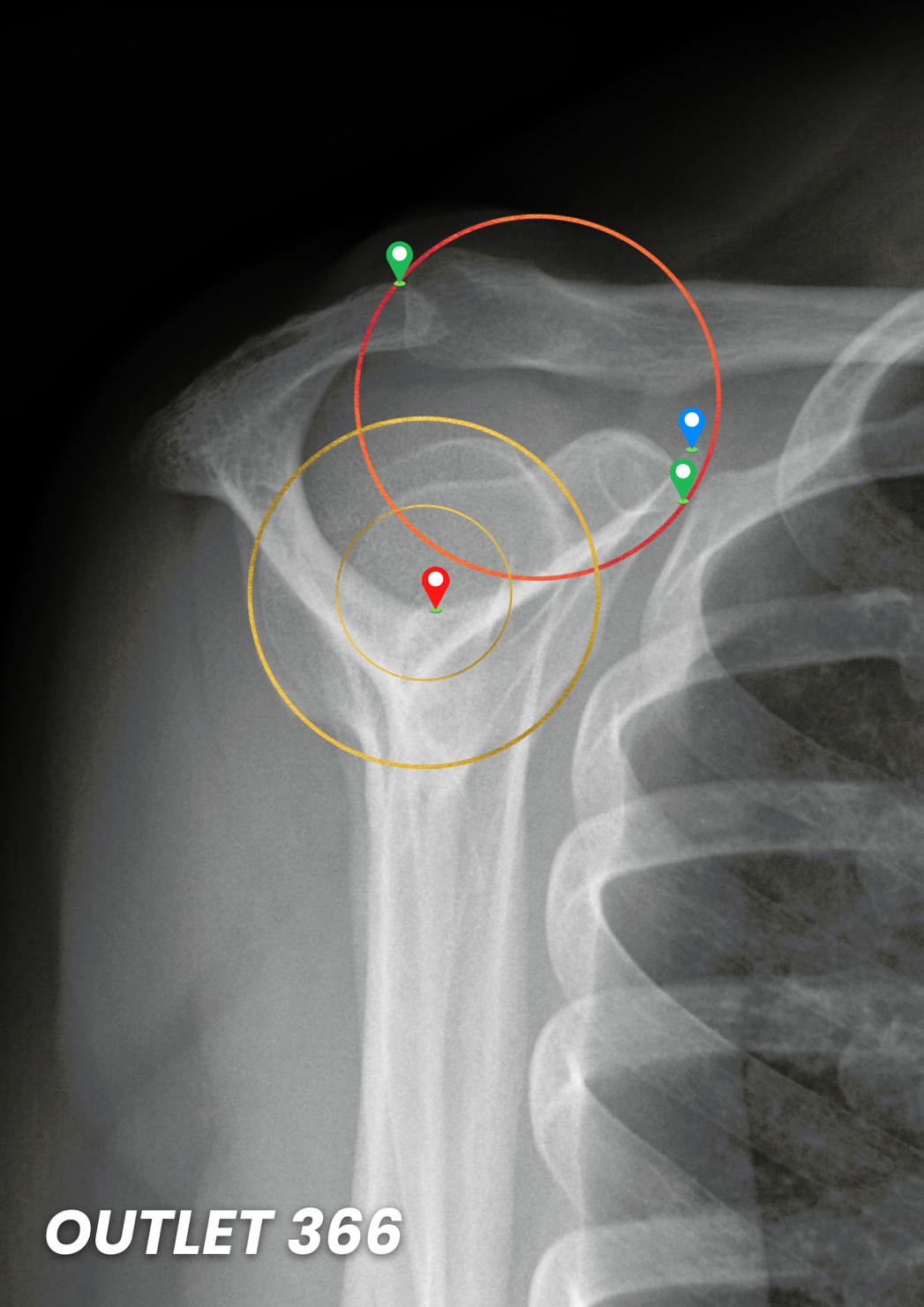


**SOV 2**

This scapula has a small amount of anterior-posterior malrotation (retroversion), which superimposes the corpus of the scapula, so that the angulus superior is moved anteriorly but just inside the AC circle.


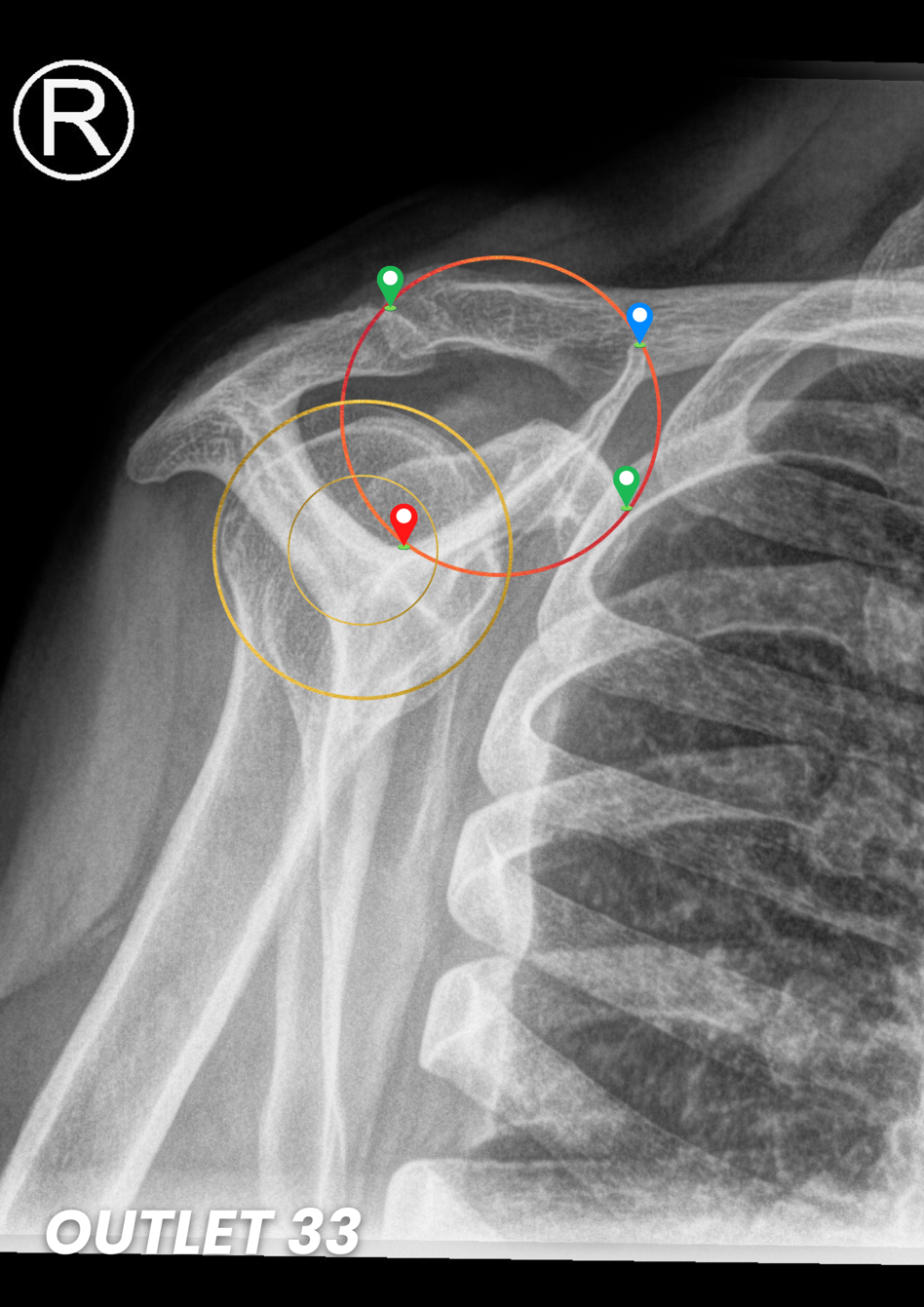


**SOV 3**

This scapula has a small combination of both anterior-posterior malrotation (retroversion) and craniocaudal malrotation (internal rotation), which both superimposes the corpus of the scapula and projects the angulus superior cranially.


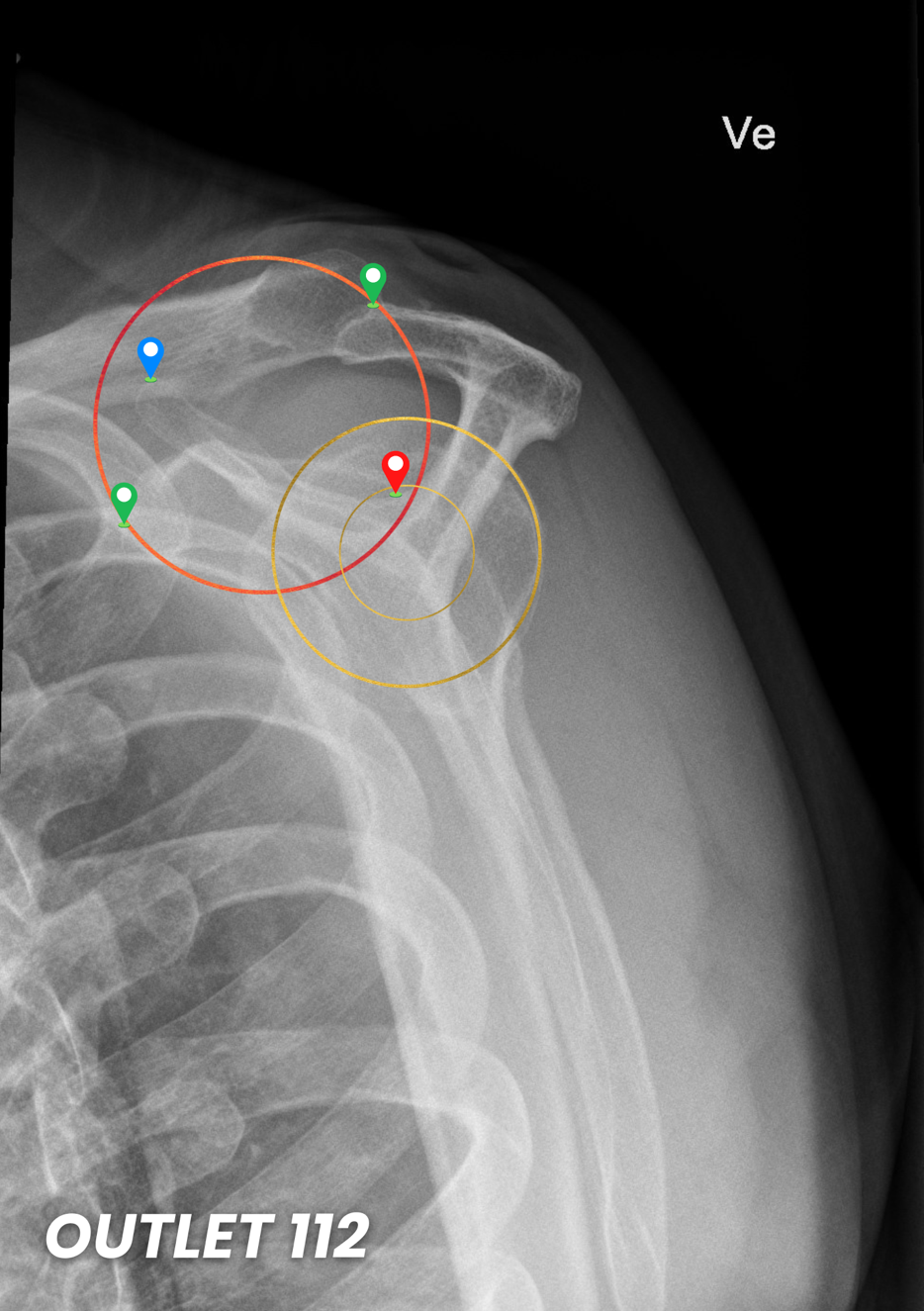


**SOV 4**

This scapula has both a combination of anterior-posterior malrotation (retroversion) and craniocaudal malrotation (external rotation), which superimposes both the corpus of the scapula and the supraspinatus fossa.
